# Developing theory-informed knowledge translation strategies to facilitate the use of patient-reported outcome measures in interdisciplinary low back pain clinical practices in Quebec: mixed methods study

**DOI:** 10.1186/s12913-020-05616-5

**Published:** 2020-08-25

**Authors:** Owis Eilayyan, Regina Visca, Diana Zidarov, Patrick Ware, André Bussières, Sara Ahmed

**Affiliations:** 1 School of Physical & Occupational Therapy, Faculty of Medicine, McGill University, 3654 Prom Sir-William-Osler, Montréal, QC H3G 1Y5 Canada; 2 College of Applied Medical Sciences, Physical Therapy and Rehabilitation Department, Jouf University, Sakaka, Jouf Saudi Arabia; 3 Center for outcome research and evaluation, Clinical Epidemiology, McGill University Health Center, McGill University, Montréal, QC Canada; 4 Centre de recherche interdisciplinaire en réadaptation (CRIR), Constance Lethbridge Rehabilitation Center, Montréal, QC Canada; 5 RUISSS McGill Centre of Expertise in Chronic Pain, Montréal, QC Canada; 6 Family Medicine, Faculty of Medicine, McGill University, Montréal, QC Canada; 7 Faculté de Médecine, École de réadaptation, Université de Montréal, Montréal, QC Canada; 8 Institut universitaire sur la réadaptation en déficience physique de Montréal, Centre intégré universitaire de santé et de services sociaux du Centre-Sud-de-l’Île-de-Montréal, Montreal, QC Canada; 9 Centre de recherche interdisciplinaire en réadaptation (CRIR), Institut de réadaptation Gingras-Lindsay-de-Montréal, Montréal, QC Canada; 10 Centre for Global eHealth InnovationCentre for Global eHealth Innovation, University Health Network, Toronto, ON Canada; 11 Département chiropratique, Université du Québec à Trois-Rivières, Trois-Rivières, QC Canada

**Keywords:** Patient-reported outcomes, Theoretical domain framework, Behavioral change techniques, Intervention mapping, Interdisciplinary team, Low back pain

## Abstract

**Background:**

There is a growing interest among healthcare providers (HCPs) to use Patient Reported Outcome Measures (PROMs) in clinical care. PROMs can help improve patient-care provider communication and may be used to inform the need for interdisciplinary care for Low Back Pain (LBP). However, PROM implementation to support clinical decision-making is complex and requires knowledge translation (KT) interventions that will overcome barriers to using PROMs in interdisciplinary clinical settings.

**Objectives:**

to 1) identify potential barriers and enablers to using PROMs in primary care LBP clinical practice from the perspective of healthcare team members, and 2) develop a theory-based tailored KT intervention to facilitate the use of PROMs in interdisciplinary clinical practice.

**Methods:**

We invited 25 HCPs working in an interdisciplinary team to complete a self-administered survey designed based on the Theoretical Domain Framework (TDF) to identify the barriers and enablers to using PROM scores in LBP clinical practice. The questionnaire consisted of 30 questions rated on a 5-point Likert scale (quantitative) and included open-ended questions (qualitative). Quantitative and qualitative data were analysed to estimate the frequency of barriers and enablers. Findings were then reviewed by a panel of four KT experts who mapped behaviour change techniques to barriers identified that informed the design of a KT intervention.

**Results:**

Eighteen HCPs responded to the survey. Factors identified as likely to restrict the use of PROM scores included *knowledge, skills, social/professional role and identity, goals, decision processes, beliefs about consequences, environmental context and resources, behavioural regulation,* and *social influence*. A multi-component evidence-based KT intervention was proposed by the panel of experts to address these barriers: a training workshop; educational materials; and use of PROM score reports to HCPs that were all delivered by an opinion leader.

**Conclusion:**

The routine use of PROMs in clinical practice may optimize the quality of LBP care and improve patients’ outcomes. The proposed multi-component KT intervention is expected to be an effective strategy to increase HCPs’ ability to integrate PROMs into clinical decision-making and to engage patients in their care.

## Background

Low Back Pain (LBP) is considered one of the highest ten conditions that cause long-term disability [1]. The estimated prevalence of LBP in industrialized countries is 60 to 70%. In Canada, the estimated direct cost of care for LBP is $6 to $12 billion every year [2]. LBP harms individuals’ physical, mental, and social activities as well as impacts on their family, society, and work-life [3–6]. The individual experience of the impact of pain varies, and as such, it is critical to evaluate impact as reported by people with LBP. Patient-Reported Outcome Measures (PROMs) are used to evaluate the impact of chronic LBP on the individuals’ function and health-related quality of life, and to evaluate the progression of LBP [7–9]. PROMs can also play a particularly important role in the management of LBP, as they can be used to screen patients for types of service needed [10].

Thus, there is a growing interest among healthcare providers (HCPs) and the broader health care system to use PROMs in clinical care [11, 12]. Patient-reported outcomes are “any report of the status of a patient’s health condition that comes directly from the patient, without interpretation of the patient’s response by a clinician or anyone else” [13]. PROMs allow HCPs to incorporate the patient voice in treatment planning and to evaluate the impact of their health condition on their function and health-related quality of life (HRQL) [14, 15].

The use of PROM scores in clinical practice can enhance the quality of patient care by influencing communication (patient-HCP, HCP-HCP, HCP-caregivers, patient-caregivers), uncovering problems experienced by patients such as psychological and functional problems, monitoring response to treatment, providing information about the impact of interventions, informing clinical decision-making [14, 16], and identifying gaps in the care currently provided [17]. Furthermore, feeding information on HRQL obtained from PROM scores back to HCPs may prompt HCP-patient discussion of HRQL issues and allow for mutual input on treatment and goal setting [18]. Such discussion is expected to enhance patient-centered care [10], and has been shown to increase patient adherence to treatment and satisfaction with care [19].

Despite the potential benefits of PROMs, there are several barriers to the routine use of PROMs. These include HCPs’ characteristics and beliefs [20–28], methodological concerns about the reliability, validity, and interpretability of PROM scores [21, 22, 29–35], feasibility or logistical issues related to implementation [21, 22, 24, 27, 36–40], and burden on patients to complete long PROMs [21].

Training HCPs to use PROMs is necessary to ensure the appropriate use of PROMs in clinical practice [41]. However, the literature shows that it is not easy to change the behaviour of HCPs in clinical practice [42, 43]. Therefore, theory-based interventions that are systematically designed to target barriers to professional behaviour change are more likely to reduce knowledge-practice gaps [44–46] and help providers implement PROMs [47–50].

The objectives of this study were to 1) identify potential barriers and enablers to using PROMs in primary care LBP practice from the perspective of LBP interdisciplinary healthcare team, and 2) develop a theory-based tailored Knowledge Translation (KT) intervention to facilitate the use of PROMs in LBP clinical practice by addressing the identified barriers.

### Conceptual framework to identify barriers to clinical behavioral change

The Theoretical Domain Framework (TDF) was used in this study to identify barriers to clinical behavioral change among HCPs and to inform the design of the theory-based KT intervention [51–55]. The TDF includes the factors that contribute to behaviour change among HCPs which are organized in the following 14 domains: Knowledge, Skills, Social/Professional Role and Identity, Beliefs about Capabilities, Optimism, Beliefs about Consequences, Reinforcement, Intentions, Goals, Memory/Attention and Decision Processes, Environmental Context and Resources, Social Influences, Emotion, and Behavioural Regulation [56].

## Methods

### Study design

We used a triangulation mixed-method design where the quantitative and qualitative data were used to understand participants’ perceptions on the use of PROM in clinical setting. The McGill University Research Ethics Board approved the study (A04-E28-16B), and all participants provided written/ electronic informed consent.

### Setting

Five primary interdisciplinary clinics located within four Health and Social Services Centres (CSSSs), and one rehabilitation center in the province of Quebec, Canada participated in the study. These five clinics were chosen as they had a LBP interdisciplinary care program and at that time this was the only program in primary care in Quebec for chronic pain aimed at supporting self-management and focused on patient-centered care. The approach being used was to assess functional, psychological and social domains. This study was an extension of ongoing work to evaluate the impact of an interdisciplinary LBP program on individuals’ health-related quality of life [57]. Improving health services at the primary care clinics through the use of best practices in LBP management may minimize unnecessary referrals to other care levels, shorten wait times, and ensure that individuals receive the care they need to address particular aspects of health and well-being (physical, mental, and development of self-management skills).

The development of the KT intervention followed a systematic framework proposed by French et al. (2012) [50], and team members have used it in prior studies [58–60]. The framework includes four key questions:

1. Who needs to do what, differently? (i.e., identify the evidence-practice gap). For this question, previous work among interdisciplinary HCPs identified the use of PROM scores in clinical practice as a gap in the delivery of a chronic pain program [61].
2. Using a theoretical framework, which barriers and enablers need to be addressed?
3. Which intervention components (behaviour change techniques and mode(s) of delivery) could overcome the modifiable barriers and enhance the enablers?

The second and third questions were addressed using two distinct phases: *phase 1* aimed to identify the key barriers and enablers to using PROMs using a self-administered survey based on the TDF. The findings of phase 1 helped develop a KT intervention to overcome the identified barriers using a panel of experts (*Phase 2*).

1. How can behaviour change be measured and understood? This question is beyond the scope of the current paper and will be the focus of future work.

### Phase 1: identifying barriers and enablers to the clinical application of PROMs

#### Participants

All twenty-five interdisciplinary HCPs, including physicians, nurses, physiotherapists, occupational therapists, kinesiologists, and psychologists, at four Health and Social Services Centres (CSSSs) and one rehabilitation center in the province of Quebec, Canada, received a self-administered survey. HCPs were eligible if they were: 1) treating individuals with LBP and, 2) fluent in English or French.

#### Survey questionnaire

A self-administered survey was developed based on the TDF [56] by the research team to explore HCPs’ perceived barriers and enablers to using PROMs. The survey included 30 questions adapted from validated TDF instruments [62, 63]. Survey items covered the 14 TDF domains, and at least two items covered each domain. Each item was rated on a 5-point Likert scale (from “Strongly agree” to “Strongly disagree”). The survey also had 16 open-ended questions to obtain more information on HCPs’ practice of using PROMs (e.g. *Are there any factors in your practice likely to help/prevent you use PRO scores in the management of patients with LBP*?). Two KT experts (AB and SA) reviewed and validated the content of a first draft of the survey in English. The survey was then translated from English into French by one fluent French/English speaker. After that, it was translated back to English by two fluent French/English speakers. Lastly, the English and French versions of the survey were reviewed by a certified translator to validate the survey. There were no significant differences between the English and French versions. Additional File 1 presents the survey items.

#### Procedure

Participants completed the survey online or if they preferred on paper. A research assistant sent a reminder to participants if they did not complete the survey within 2 weeks. The survey took approximately 20 min to complete.

#### Data analysis

##### Quantitative data

Data from the survey were descriptively analyzed. The responses to each item were categorized into “agree/strongly agree” and “disagree/strongly disagree/neutral.” The former category referred to the enablers, while the latter referred to the barriers. The percentage of participants who chose “agree/strongly agree” and “disagree/strongly disagree/neutral” was calculated to determine if the construct represented by the survey item was a barrier or an enabler. The items were ranked based on the proportion of “Strongly disagree/ disagree/ neutral” responses from the highest to the lowest percentage. In this study, an enabler and barrier was defined as an item that had > 60% of respondents answering “agree/strongly agree” and “strongly disagree/ disagree/ neutral”, respectively. There is no evidence defining a cutoff point in order to determine the barriers and enablers of using evidence-based practice (EBP). Thus, we used 60% as a cutoff point, indicating that a clear majority of participants experienced the barriers to implementing the PROMs.

The sample size needed to answer the survey’s question was calculated, based on population size, confidence interval level, and margin error following the equation: (Z^2^*p(1-p)/e^2^) / 1 + (Z^2^*p(1-p)/e^2^N) [64]. At 95% of confidence interval and 5% of error, the sample size needed was 24 subjects.

##### Qualitative data

Content analysis was used to analyse the open-ended question data where the frequency of keywords was counted to determine the main themes from the data. Numerous studies used content analysis to analyse open-ended questions/comments [65–69]. The first author (OE) reviewed the answers of participants and identified the keywords. In some cases, the responses of the question could have more than one keyword. The analysis was completed in two phases: inductive and deductive analysis [65]. Inductive analysis: the keywords from participants’ responses were identified (participants coded). Then, the percentage of agreement on keyword selection among participants was determined; number of participants coded divided by the total number of participants who responded to each question. The deductive analysis: the keywords emerged from the first part were categorized into barriers and enablers, and then mapped into the TDF framework.

Both data from the open and closed-ended questions was triangulated to identify the barriers and enablers for using PROMs and informed the intervention design to facilitate the use of PROMs n clinical care.

### Phase 2: intervention design

This phase aimed to design a theory-based tailored KT intervention to address previously identified barriers using intervention mapping.

#### Participants & procedure

The KT intervention was designed by a panel of experts that included six health service and KT experts including a knowledge broker that worked with the clinics (AB, SA, RV, AG, DZ, and OE) who were familiar with TDF and behavioral change techniques (BCTs). The experts reviewed the barriers identified in phase 1 and considered more than 100 evidence-based BCTs listed in Michie et al. studies [70, 71]. Then, experts mapped the key barriers to the most appropriate BCTs. Subsequently, they brainstormed to identify the most suitable KT interventions with evidence supporting its effectiveness to change professional behaviours and the modes of delivery of the intervention to address each of the key barriers. Finally, experts reached consensus over the BCTs and the modes of delivery to recommend based on supporting evidence [72] and feasibility to be implemented in the respective clinical settings [73]. A BCT is “an observable and replicable component designed to change behaviour” [73]. Delineating BCTs is needed to select appropriate behavior change strategies for the implementation of PROMs and evaluation of the proposed KT intervention [73].

## Results

### Phase I: self-administered survey

#### Characteristics of participants

Eighteen HCPs completed the survey (response rate of 72%), including two physicians, six physiotherapists (PTs), three nurses, three psychologists, two occupational therapists (OTs), and two kinesiologists. The mean age of the participants was 39 years (SD ± 7.7); 39% (7/18) were females, and the mean number of years in practice was 14 years (SD ± 8.4). Table 1 shows the characteristics of participants.

**Table 1:** Characteristics of Participants

Characteristic	M (sd) / N (%)
Age	39 (7.7)
Gender, % Female	7 (39%)
Education level	
Bachelor	6 (33%)
Master	8 (44%)
Doctorate	2 (11%)
Profession	
Physician	2 (11%)
Nurse	3 (17%)
Physiotherapy	6 (33%)
Psychologist	3 (17%)
Kinsiologist	2 (11%)
Occupational Therapy	2 (11%)
Years of experience	14 (8.4)

#### Key barriers and enablers in the self-administered survey

##### Quantitative data

Additional File 2 presents the responses to the PROM self-administered survey, and Table 2 presents a summary of the enablers and barriers. The close-ended questions revealed ten enablers to use PROMs that were mapped onto 8 TDF domains: *knowledge (72%), skills (61%), social/professional role and identity (83%), optimism (72%), beliefs about consequences (72%), reinforcement (67%), intentions (78%), and emotion (78%)*.

**Table 2:** Key TDF enablers and barriers domains to use PROMs among interdisciplinary healthcare team

TDF Domain	Close-ended Question	Enabler/Barrier
Knowledge	Awareness of the objectives of using PROMs in clinic: **13 (72%)**	Enabler
Skills	Having the skills needed to interpret the results of PROMs: **11 (61%)**	Enabler
Skills	New skills are required to successfully use PROMS in the management of patients with LBP: **15 (83%)**	Barrier
Social/Professional Role and Identity	Believing that using PROMs is one of the HCPs’ role in clinic for individual patient management of LBP: **15 (83%)**	Enabler
Social/Professional Role and Identity	Professional role as HCPs in using PROMs in clinics was not clear: **15 (83%)**	Barrier
Optimism	Expecting improved patient outcomes as a result of using PROMs in the management of patients with LBP: **11 (61%)**	Enabler
Optimism	Optimism regarding the benefits of using PROMs in the management of patients with LBP: **13 (72%)**	Enabler
Beliefs about Consequences	Believes in the benefits of using PROMs in the management of patients with LBP: **13 (72%)**	Enabler
Beliefs about Consequences	Using PROMs in clinical practice is not necessary to improve patient outcomes: **12 (67%)**	Barrier
Reinforcement	Having better patient health outcomes makes HCPs continue using PROMs in the management of patients with LBP: **12 (67%)**	Enabler
Intentions	HCPs’ commitment to use PROMs in the treatment of patients with LBP in the next three months: **12 (67%)**	Enabler
Intentions	Having a strong intention to use PROMs in the treatment of patients with LBP in the next three months: **14 (78%)**	Enabler
Goals	The use of PROMs in the treatment of patients with LBP is not more important and prioritized compared to only using clinical outcomes: **15 (83%)**	Barrier
Goals	The plan of how to use PROMs in clinical practice is not clear: **10 (56%)**	Barrier
Decision Processes	The use PROMs is difficult in making treatment decisions: **11 (61%)**	Barrier
Environmental Context and Resources	Lack of time to use PROM scores in the clinical setting: **12 (67%)**	Barrier

*LBP* Low Back Pain*, PROMs* Patient Reported Outcome Measures*, TDF Theoretical Domain Framework*

In the survey’s close-ended questions, eight barriers that corresponded to eight TDF domains were identified; more than 60% of the participants considered them as barriers to implement PROMs. The domains included *skills (83%), social/professional role and identity (83%), goals (83%), decision processes (61%), beliefs about consequences (67%), environmental context and resources (67%), behavioural regulation (67%),* and *social influence (83%)*. Also, four TDF domains had a high percentage of “neutral” responses (*knowledge*, *beliefs about capabilities*, *memory*, and *reinforcement*); these latter domains were classified as barriers. Table 2 shows the barriers and enablers based on the close-ended questions.

##### Qualitative data

This section presents examples of the data extracted from the open-ended questions included in the survey.

*“****What information do you believe is necessary for a clinician to be able to use PRO scores in the management of patients with LBP?”***

Fourteen participants responded to this question. Six participants (43%) stated that they needed the PROM scores interpretation to be able to use it in the clinical setting. Two participants (14%) mentioned that presenting the psychometric properties of PROMs and providing compiled PROM score results were necessary information.

“***What new skills do you feel you need to acquire to be able to use PRO scores for individual patient management of LBP?”***

Ten participants responded to this question, seven participants (70%) agreed that having the knowledge and skills to interpret PROM scores is required for clinicians to be able to use PROMs in clinical care for management of LBP.

***“What are the benefits of using PRO scores for patient management of LBP?”***

Ten participants responded to this question, three participants (30%) acknowledged that using PROMs helps with understanding patients’ perceptions of their condition and two participants (20%) mentioned that using PROMs makes the evaluation of patients faster. Other advantages that were listed included: facilitating communication between HCPs and patients, monitoring patients over time, evaluating and modifying the treatment, and using PROM scores for clinical decision-making. Lastly, one participant stated that using PROMs in clinics to show patients their changes in scores might increase their motivation to adhere to the treatment.

***“What are the potential disadvantages of using PRO scores in the management of patients with LBP?”***

Eleven participants responded to this question. Two participants (18%) indicated that using PROMs did not provide a full explanation of a patient’s problems. Also, two participants (18%) mentioned that using PROMs requires a lot of time for a patient to complete. Other disadvantages of using PROMs included patients’ difficulties in understanding some PROM questions, too much data to manage, and discrepancies between PROM results and clinical observations.

***“Are there any factors (e.g. motivation, availability of patients’ scores, enough time, etc…) in your practice likely to help you use PRO scores in the management of patients with LBP?”***

Twelve participants responded to this question. Six participants (50%) stated that the availability of and access to patients’ scores helped use PROMs in the clinical setting, which is mapped into “***environmental context and resources***” of the TDF. In addition, the following facilitation factors were mentioned twice (17%): having time to use PROMs, compilation of scores, patients’ and HCPs’ motivation to use PROMs. Furthermore, other factors were also listed as facilitators to use PROMs: understanding patients scores, training on PROM scores interpretation, teamwork, and having patients fill out the questionnaires electronically. Table 3 presents the facilitators that emerged from this question.

***“Are there any factors (e.g. lack of knowledge, lack of time, lack of access to patients’ scores, etc…) in your practice likely to prevent you from using PRO scores in the management of patients with LBP?”***

Thirteen participants responded to this question, six participants (46%) stated that lack of knowledge of use PROMs and lack of time were factors to restrict the use of PROMs. These two factors mapped onto “***knowledge***” and “***environmental context and resources”*** TDF factors, respectively. Three participants (23%) mentioned that lack of scores interpretation restricted the use of PROMs. Lastly, lack of resources to compile patient data, availability of validated French-Canadian questionnaires and access to patient results were considered as barriers to use PROMs. Table 4 presents the barriers emerged from the question.

“***How easy or difficult is using PRO scores in the management of patients with LBP? What problems or barriers have you encountered using PRO scores for the management of patients with LBP?”***

Fourteen participants responded to this question. Four (29%) participants acknowledged that using PROMs in the management of patients with LBP was difficult while two (14%) participants said it was easy. In addition, five (36%) participants mentioned that using PROMs took time to complete, and two participants (14%) said that lack of interpretation of PROM scores was a barrier. Furthermore, two participants (14%) stated that PROM data were out of date if taken too long before the clinic visit. Other barriers included lack of knowledge and resources to compile data. Lastly, one participant mentioned that PROMs allow a deep clinical analysis.

**Table 3:** “Are there any factors (e.g. motivation, availability of patients’ scores, enough time, etc…) in your practice likely to help you use PRO scores in the management of patients with LBP?”

Facilitator	Agreement Percentage	TDF factor
Availability of and access to patients’ scores	50%	***Environmental context and resources***
Having time to use PROMs	17%	***Environmental context and resources***
Compilation of scores	17%	***Environmental context and resources***
Patients’ and HCPs’ motivation to use PROMs	17%	***Reinforcement***
Understanding patients scores	8%	***Knowledge***
Training on PROM scores interpretation	8%	***Skills***
Teamwork	8%	***Environmental context and resources***
Having patients fill out the questionnaires electronically	8%	***Environmental context and resources***

**Table 4:** “Are there any factors (e.g. lack of knowledge, lack of time, lack of access to patients’ scores, etc…) in your practice likely to prevent you using PRO scores in the management of patients with LBP?”

Barriers	Agreement Percentage	TDF factor
Lack of knowledge of use PROMs	46%	***Knowledge***
Lack of time	46%	***Environmental context and resources***
Lack of scores interpretation	23%	***Skills***
lack of resources to compile patient data	8%	***Environmental context and resources***
Lack availability of validated French-Canadian questionnaires	8%	***Environmental context and resources***
Lack of access to patient results	8%	***Environmental context and resources***

#### Final list of barriers and enablers

The integration of open and close-ended response data resulted in nine barriers that corresponded to nine TDF domains: *knowledge*, *skills, social/professional role and identity, goals, decision processes, beliefs about consequences, environmental context and resources, behavioural regulation,* and *social influence*. On the other hand, both open and close-ended response resulted in eleven enablers to use PROMs that were mapped onto 9 TDF domains: *knowledge, skills, social/professional role and identity, optimism, beliefs about consequences, reinforcement, intentions, emotion, and environmental context and resources.*

### Phase II: intervention design

Table 5 presents the details of the mapping of BCTs onto key barriers identified. This section presents BCTs and intervention components selected by KT experts to address those key barriers aforementioned (Phase 1; *knowledge, skills, social/professional role and identity, goals, decision processes, beliefs about consequences, environmental context and resources, behavioural regulation,* and *social influence*), based on current evidence and feasibility to implement strategies within the clinical settings (see Additional File 3 for details). The four main KT intervention components were:

1. Educational/ instructional material on the selection, application, and interpretation of the PROM scores and the HCPs’ roles in using PROMs to plan treatment and monitor changes in outcomes in collaboration with patients.
2. A half-day training workshop on the use of PROMs in clinical practice (see Additional file 3 for details).
3. Feedback reports of individuals’ PROM scores (Additional File 3); and
4. The use of an opinion leader to support the implementation of the KT intervention components and to provide coaching to HCPs on the use of PROMs. Opinion leaders are a known enabler of implementation and defined as “Individuals in an organization who have formal or informal influence on the attitudes and beliefs of their colleagues with respect to implementing the intervention” [74].

**Table 5:** Mapping behavior change techniques on identified barriers, suggested KT intervention components and delivery methods

Step 1: Review the Barriers	Step 2: Mapping the barriers to TDF domains	Step3: Mapping the barriers on BCTs	Step 4: Proposed all possible interventions	Step 5: Selected the appropriate interventions
Lack of knowledge and skills, new skills needed to acquire to use PROMs in clinics - ability to score and interpret the PROM data - link the scores to the patient prognosis Aim: to improve the HCPs’ skills in scoring and interpreting PROMs data and using it in the clinical practice.	**Knowledge/ Skills**	• Self-monitoring; • Monitoring; • Reward/Incentives • Graded tasks (start easy); • Increasing skills (problem solving; decision making, goal setting); • Rehearsal of relevant skills; • Modeling, demonstration of behavior by others; • Homework	• Continuing education: offer HCPs courses on pain management that emphasize the importance of using PROMs in clinical setting. • Workshop: educational and training workshop (Interactive workshops) in small groups led by the research team to coach the HCPs on using PROMs in clinical practice. • Distributing educational/instructional materials to the HCPs to improve their skills on LBP PROM scores interpretation, understanding the minimal clinically important change, and linking it to the treatment components that should be implemented • Webinar and video to provide information about scoring and interpreting a wide range of PROMs in LBP area. • Mass Media: social media to provide information about scoring and interpreting a wide range of PROMs in LBP area • Opinion Leader: identifying and training an opinion leader in each clinic to facilitate the using of PROMs (one to one coaching) • Educational outreach visits: send a trained person (e,g, champion clinician) to the clinic to coach, monitor, and provide feedback on PROM use to HCPs in person.	✓
				✓
				✓
Lack of professional role clarity in using PROMs - the role of HCPs in using PROMs in clinical practice is not clear Aim: to introduce and instruct the HCPs about how PROMs relate to their roles in the clinical setting.	**Social/Professional Role and Identity**	• Social processes of encouragement, pressure, and support; • Instruction; • Persuasive communication	• Workshop: educational workshop (interactive workshop) to introduce participants on the clinician’s role about the use of PROMs within their clinical practice. • Opinion Leader: identifying and training an opinion leader in each clinic to facilitate the using of PROMs and to deliver the key messages to the HCPs (i.e. HCPs’ role). • Distributing educational materials to the HCPs on their role in using PROMs score within their clinical setting. • Educational outreach visits: send a trained person (one of the research member) to the clinic to give information and feedback on the PROMs to the HCPs • Webinar to provides information about the roles of HCPs in using PROMs	✓
				✓
				✓
It is not clear how to use PROMs in the clinical setting. - the implementation process of using PROMs in the clinical practice is not clear for some HCPs Aim: to clarify the implementation process of PROMs	**Goals**	• Behavioral information; • Social processes of encouragement, pressure, and support; • Increasing skills (problem solving; decision making, goal setting); • Reward/Incentives; • Motivational Interviewing	• Developing and using PROM scores reports that includes the patients’ scores, interpretation, and the treatment algorithm (first proposed action plan) • Developing and implementing an electronic platform that includes the patients’ scores, interpretation, and the treatment algorithm (alternative proposed action plan) • Workshop: introduce and coach the HCPs on the PROMs scores report and/or the electronic platform • Distributing instructional materials explaining the use of the proposed PROM scores report or electronic platform. • Opinion Leader: identifying and training an opinion leader in each clinic to facilitate the using of PROMs and help the HCPs to use the scores report and/or electronic platform. • Educational outreach visits: send a trained person (one of the research member) to the clinic to coach the HCPs in person to implement PROMs using scores report/electronic platform. •Webinar to provide information on the scores reports and electronic platform.	✓
				✓
				✓
				✓
Lack of priority to use PROM tools - using PROMs in clinical practice is not a priority for HCPs to support clinical decision making Aim: to encourage use of PROM scores to support clinical decision making	**Goals**	• Behavioral information; • Social processes of encouragement, pressure, and support; • Increasing skills (problem solving; decision making, goal setting); • Reward/Incentives; Motivational Interviewing	• Workshop presents the benefits and advantages of using PROM scores in the clinical setting • Distributing educational material presenting the benefits and the consequences of using PROMs in the clinical practice • Opinion Leader: identifying and training an opinion leader in each clinic to facilitate the using of PROMs and introduce the advantages of using PROMs • Educational outreach visits: send a trained person (one of the research member) to the clinic to discuss the advantages and the importance of using PROMs in clinical practice • Webinar to present the advantages and the importance of using PROMs in clinical practice • Feeding back the HCPs the outcome of their own use of PROMs (i.e. changes in patient outcomes after using PROMs)	✓
				✓
				✓
				✓
It is difficult to use PROMs in making treatment decision - using PROMs for the clinical decision making is difficult Aim: to facilitate using use of PROM scores in the clinical practice in order to use for support clinical decision making	**Decision Processes**	• Self-monitoring; • Planning/implementation; • Prompts/Triggers/Cues	• Using PROM scores report that includes the patients’ scores, interpretation, and the treatment algorithm options. Present an action plan to ease the use of PROMs in clinical decision making • Implementing an electronic platform includes the patients’ scores, interpretation, and the treatment algorithm (alternative action plan) • Workshop: introduce and coach the HCPs on the scores report and the electronic platform in making treatment decision	✓
				✓
			• Opinion Leader: identifying and training an opinion leader in each clinic to facilitate the using of PROMs and help the HCPs use the scores report/electronic platform in making treatment decision • Educational outreach visits: send a trained person (one of the research member) to the clinic to coach the HCPs in person to use scores report/electronic platform in making treatment decision • Webinar to provide information on the proposed scores reports and electronic platform and how to use them in making treatment decisions	✓
Using PROMs in clinical practice is not necessary to improve patient health outcome - Using PROMs in clinical practice is not necessary to improve patient health outcome and/or to improve the quality of patient’s care Aim: to increase HCPs’ knowledge regarding the benefits of using PROMs in clinical practice.	**Beliefs about Consequences and** ***Reinforcement***	• Persuasive Communication; • Information regarding behavior outcome; • Feedback; • Self-monitoring; • Reward/Incentives)	• Workshop that presents the benefits of using PROM scores in the clinical setting • Distributing educational material on the benefits and the consequences of using PROMs in clinical practice • Opinion Leader: Identifying and training an opinion leader in each clinic to facilitate the using of PROMs and introduce the advantages of using PROMs • Educational outreach visits: send a trained person (one of the research member) to the clinic to discuss the advantages and the importance of using PROMs in clinical practice • Webinar to present the advantages and the importance of using PROMs in clinical practice • Feeding back the HCPs the outcome of their own using of PROMs	✓
				✓
				✓
				✓
Lack of time - lack of time among HCPs is a barrier to use PROMs in clinical practice Aim: developing a tool to facilitate and maximize using PROMs in clinical setting without time consuming	**Environmental Context and Resources**	• Environmental Changes; • Time management	• Using PROM scores report that includes the patients’ scores, interpretation, and the treatment algorithm. This the first proposed plan to facilitate using PROMs in making treatment decision • Implementing an electronic platform along with the patients’ scores, interpretation, and the treatment algorithm (alternative proposed plan)	✓
HCPs don’t assess patients’ motivation to complete PROMs - patients’ motivation to use PROMs in clinical practice is not assessed by HCPs Aim: developing an assessment tool to assess the patients’ motivation to use PROMs in the clinical practice as a stakeholder. This tool is administered at the first appointment only.	**Behavioral Regulation**	• Monitoring; Environmental Changes; • Prompt	• Developing measurement tools to assess patients’ motivation to comply with completing PROMs • Providing HCPs with the motivational assessment tool to evaluate patient motivation.	✓
				✓
HCPs don’t use PROMs automatically - using PROMs in clinical practice is not easily integrated into routine clinical practice by HCPs Aim: helping HCPs to integrate and use PROMs in routine clinical practice	**Behavioral Regulation and** ***Memory***	• Self-monitoring; Contract; • Planning/implementation; • Prompts/Triggers/Cues; • Use of imagery	• Using PROM scores report that includes the patients’ scores, interpretation, and the treatment algorithm. This the first proposed plan to facilitate using PROMs in making treatment decision • Sending the HCPs a reminder to use PROMs in the clinical setting, through ▪ E-mail ▪ Decision support tool, or ▪ Opinion leader	✓
				✓
Patients influence clinician PROMs practice - patient’s view could influence their clinical practice in terms of using PROMs in the management of patients with LBP Aim: increasing the confidence of HCPs to use PROMs in the management of patients with LBP	**Social Influence**	• Social processes of encouragement, pressure, and support; • Social Support; • Modeling/ Demonstration of behavior by others	• Identifying and training an opinion leader in each clinic to facilitate the using of PROMs and to deliver the key messages to the HCPs • Providing the HCPs with the effectiveness of using PROMs to be able to provide the patients with the PROMs effectiveness in clinical practice • Workshop: educational and training workshop (Interactive Workshop) led by the research team to coach the HCPs in a group on using PROMs in clinical practice • Encouraging collaborations between interdisciplinary team members to discuss the implementation of PROMs and the benefits of using them in practice • Social media to provide information on the advantages of PROMs, with testimonials from HCPs and patients	✓
				✓
				✓

*BCTs* Behavioral Change Techniques*, KT* Knowledge Translation*, LBP* Low Back Pain*, PROMs* Patient Reported Outcome Measures*, TDF* Theoretical Domain Framework

HCPs will be introduced to the educational materials and a PROM scores report during the training workshop led by the research team members and opinion leaders. The educational materials will summarize the roles of HCPs in the use of PROM scores in the clinical setting and highlight the potential advantages of using PROMs in clinical practice. In addition, these materials will inform HCPs on how to interpret PROM scores while considering minimally clinically meaningful change over time, and provide case studies of how to link PROM scores to treatment and referral to services. During the training, the HCPs will also train on how to integrate the PROM scores with other clinician-reported information. The training workshop will use small group methodology. Interactive exercises will aim to help HCPs to practice and develop new skills.

The PROMs feedback report aims to facilitate the routine use of PROM scores in clinical settings, where HCPs receive the report before the patient visit; use scores to set objectives and a treatment plan; and facilitate shared clinical decision-making in collaboration with patients. The PROMs report presents patients’ scores over time, interpretation of scores, and the treatment algorithm. Lastly, participants will be given interactive exercises during the workshop to interpret different PROM scores and use each to make treatment decisions.

Opinion leaders with the support of research team members will lead the workshop. In this study, the clinical manager of each site was identified as the opinion leader. Before the HCPs’ workshop, opinion leaders will attend a training session where they will learn practical strategies for delivering the key messages to the HCPs (i.e., their roles, the advantages of using PROMs), strategies to support the HCPs to use PROMs in clinical practice, and encourage the collaboration between interdisciplinary team members. Furthermore, as part of the PROM implementation process, weekly automated email reminders will be sent to HCPs to use PROMs in the clinical setting with useful tips to reinforce the appropriate use of PROMs.

## Discussion

Using PROMs in clinical practice may improve the quality of healthcare services [10, 14, 16–19]. This study identified potential barriers to the use of PROMs in a primary care LBP interdisciplinary clinical setting. These barriers map to the TDF domains of *skills, social/professional role and identity, goals, decision processes, beliefs about consequences, environmental context and resources, behavioural regulation,* and *social influence*. Individuals with experience in developing KT interventions selected KT intervention components in order to address the identified barriers. The proposed intervention components were selected based on the evidence [72] and feasibility to be implemented in the respective clinical settings.

The results suggest that there are opportunities for HCPs participating in this study to further develop their skills as it relates to the use of PROMs in clinical practice, especially when it comes to scoring and interpreting PROM scores. Lack of experience in using PROMs [20] and difficulties in interpreting PROM scores have also been noted elsewhere [22, 75]. Furthermore, the participants in this study indicated having difficulty using PROMs to make clinical decisions and had no definite plans about how to use them in their clinical setting. These findings are also in line with those from previous studies that found HCPs did not know how to respond to patients’ issues identified by PROMs [23] and raised concerns that using PROMs may force patients to discuss areas about which the clinician has received little training or has little control over [27]. Other concerns identified in the literature included not having the results of PROMs at the time of consultation or follow-up appointments [38], and HCPs felt that the data from PROMs lacked clinically meaningful analyses and recommendations [21]. However, the literature showed that the combination of treatment guidelines, clinical experience of HCPs, and PROMs could support clinical treatment decisions [54, 76–78]. The process used in this study to match barriers to potential strategies can help clinical teams develop an informed plan for using PROM scores to support clinical decisions with recommendations for interventions and services that match the specific context of a clinical setting or health region.

In contrast, some participants in this study stated that using PROMs was not necessary to improve patients’ health outcomes. These findings were also found by Chang 2007 [21]. In another study, HCPs felt that using PROMs data in clinical settings was not relevant and did not help their practice [79]. In addition, the participants in the current study did not prioritize the use of PROMs for treatment decision-making, and they preferred to obtain information from peers and patients. The literature supports this as HCPs reported that information collected informally was ‘superior’ to the standard assessment tools [24], and PROMs did not provide clinically relevant results [36, 37]. Thus, increasing knowledge about the potential benefits of PROMs in clinical practice is essential, as they are the only standardized measure of patients’ reports of the impact of their health on relevant physical, social, emotional, and mental health domains. As clinical measures (e.g., blood pressure) are used to monitor the impact of treatments, PROMs are needed to ensure care is patient-centered and guided by outcomes meaningful to consumers of healthcare.

Furthermore, some HCPs in this study found that they cannot easily integrate PROMs into their clinical workflow, and HCPs also stated that lack of time was a barrier to the use of PROMs. In part, this may be related to difficulty integrating PROMs scores with other clinical data [22]. Johnston et al. (2005) found that “resource constraints,” including lack of time among HCPs, was the main barrier to using PROMs data in routine clinical practice [80]. Similarly, another study conducted in 2004 found that psychologists in clinical practice stated that using PROMs data took too much time [79]. Despite this, two studies showed that using PROMs in clinical practice did not increase the therapeutic session duration [81, 82]. An essential component of training will be the joint interpretation of PROM scores with other clinician-reported information and identifying *who* (patient, HCPs, administrators) needs to do *what* and *when* to ensure PROMs are collected and scores are available during the clinical encounter. Each of these aspects will likely facilitate the integration of PROMs into the clinical workflow.

Several enablers were identified by study participants that could help bolster the use of PROMs in clinical practice and enhance the KT intervention that aimed to support the use of PROMs. For example, the participants showed a high intention to use PROMs, unlike the results of previous studies that found a lack of motivation among HCPs for using PROMs in clinical practice [26]. Also, almost half of the participants stated that they had the self-confidence to use PROMs data in the clinical setting, which is in contrast with the results of a previous study that found a lack of confidence in using PROMs in a lung transplant department [49]. This difference might be because of the environmental context of the participating clinics in our study, where there were strong collaborations between HCPs and support from managers. Lastly, HCPs in this study stated that they would keep using PROMs in clinical practice if it can help support clinical care decisions and improve patients’ health outcomes. The operant learning theory supports this; the achievements of a behavior determine the continued use (i.e. reinforcement) of that behavior in the future [54].

Several components of KT intervention based on BCTs were proposed by the expert panel to address the identified barriers to using PROM scores. The main KT intervention components selected based on the evidence and feasibility included educational and training workshops, standardized printed materials (PROM scores report), educational/instructional materials, and the support of an opinion leader. The proposed KT intervention components will form a multi-component intervention, which can address a more significant number of gaps and barriers [83], increase HCPs’ knowledge, and foster best practices The specific PROMs selected for interactive exercises and the implementation process of PROMs will be adapted for each clinical site receiving the training.

The evidence for the effectiveness of these approaches supports our KT intervention. Two high-quality reviews showed that providing educational materials to healthcare providers has a significant effect on improving their practice [84, 85]. The educational materials help change the beliefs of HCPs towards the implementation of evidence-based practice [86], which in turn may facilitate the use of PROMs in clinical practice.

Furthermore, another high-quality review supported the effectiveness of educational workshops by; “implementing educational meetings, either alone or combined with other interventions, significantly improved the HCPs’ practice in the clinical setting” [87]. On the other hand, three high-quality reviews demonstrate conflicting results of the effectiveness of educational meetings on HCPs’ practices [88–91]. Hatfield & Ogles 2004 showed that conducting training among HCPs to use PROMs data had a significant positive effect on participants’ attitude and behaviour [79]. Lastly, Flodgren, et al. review found that an opinion leader intervention, used alone or combined with other interventions, improves HCPs’ practices [92].

The proposed theory-based KT intervention that was developed in this study may empower HCPs to change their practices, allowing them to use PROMs for clinical decision making confidently. In turn, HCPs, in collaboration with patients, may optimize the use of health care services by matching individuals with LBP to interventions and programs based on their needs, and consequently, reduce pain interference, maximize functional ability and quality of life, and decrease cost burden [93–95].

### Strengths and limitations

To our knowledge, this is the first study aimed at developing KT strategies guided by theory to facilitate the application of PROMs in clinical practice in interdisciplinary LBP clinical programs according to interdisciplinary HCPs’ perspectives. The KT intervention developed in this study is a theory-based design using a systematic approach informed by an expert panel, which strengthened the selection of the intervention components. However, one limitation is that patient representatives were not involved in the expert panel.

Five sites and 18 healthcare providers working in an interdisciplinary program participated in this study, therefore, generalization of the quantitative findings may be limited. However, due to the theory-driven nature of this research (i.e., TDF used extensively across different populations, health disorders, context and settings), this study may act as a widely applicable model for assessing barriers to the use of PROMs by HCPs and developing tailored and evidence-based KT interventions aimed at optimizing their uptake and regular use. Future work using the process outlined in this study will provide information about whether the same barriers and enablers emerge.

The reliability and validity of the survey used in this study were not assessed, however, the items were derived from reliable and valid surveys based on the TDF [62, 63].

## Conclusion

The key TDF domains likely to influence the use of PROMs included clinician *knowledge, skills, social/professional role and identity, goals, decision processes, beliefs about consequences, environmental context and resources, behavioural regulation,* and *social influence*. Mapping these domains to BCTs resulted in a multicomponent KT intervention supported by current evidence and considered feasible to overcome barriers and maximize the application of PROMs among HCPs in LBP clinics. The effectiveness of the proposed KT intervention in changing HCPs’ behaviour toward the delivery of self-management support will be evaluated in a cluster randomized controlled trial.

## Supplementary information

**Additional file 1.** PROMs Self-administered Survey Output.

**Additional file 2.** PROMs Survey.

**Additional file 3.** Patient Reported Outcome Measures (PROMs) KT intervention.

## Data Availability

The datasets used and/or analysed during the current study are available from the corresponding author on reasonable request.

## Abbreviations

BCTs: Behavioral Change Techniques
CSSSs: Health and Social Services Centres
HRQL: Health-related Quality of Life
KT: Knowledge Translation
LBP: Low Back Pain
PROMs: Patient Reported Outcome Measures
TDF: Theoretical Domain Framework
