# Supplementary material for: Developing theory-informed knowledge translation strategies to facilitate the use of patient-reported outcome measures in interdisciplinary low back pain clinical practices in Quebec: mixed methods study
Source: BMC Health Serv Res. 2020 Aug 25;20:789. doi: 10.1186/s12913-020-05616-5 (PMC7445906; doi:10.1186/s12913-020-05616-5)
Supplement: Supplementary file 1 — Additional file 1. PROMs Self-administered Survey Output. [file 12913_2020_5616_MOESM1_ESM.docx]

| Additional File 1: PROMs Self-administered Survey Output | | | | | |
| --- | --- | --- | --- | --- | --- |
| Question | **Strongly Agree** | **Agree** | **Neutral** | **Disagree** | **Strongly Disagree** |
| Knowledge | | | | | |
| 1- I am aware of the objectives of using PRO scores in the management of patients with LBP | 4 (22%) | 9 (50%) | 5 (28%) |  |  |
| 2- I have sufficient knowledge about how to apply PRO scores in the management of patients with LBP | 2 (11%) | 6 (33%) | 7 (39%) | 2 (11%) | 1 (6%) |
| 3- I know how to interpret PRO scores in the management of patients with LBP | 1 (6%) | 8 (44%) | 6 (33%) | 2 (11%) | 1 (6%) |
| Skills | | | | | |
| 4- I have the skills to interpret PRO scores in the management of patients with LBP | 2 (11%) | 9 (50%) | 6 (33%) |  | 1 (6%) |
| 5- I need to acquire new skills to be able to use PRO scores in the management of patients with LBP (rescaled) |  | 3 (17%) | 4 (22%) | 8 (44%) | 3 (17%) |
| Social/Professional Role and Identity | | | | | |
| 6- I think my role as …… should include using PRO scores for individual patient management of LBP | 3 (17%) | 12 (75%) | 2 (11%) | 1 (6%) |  |
| 7- My role in using PRO scores in the management of patients with LBP are clearly defined for me | 1 (6%) | 2 (11%) | 6 (33%) | 7 (39%) | 2 (11%) |
| Beliefs about Capabilities | | | | | |
| 8- I am confident that I can use PRO scores in the management of patients with LBP | 1 (6%) | 9 (50%) | 5 (28%) | 2 (11%) |  |
| 9- I am comfortable with using PRO scores in the management of patients with LBP | 1 (6%) | 5 (28%) | 11 (61%) |  | 1 (6%) |
| Optimism | | | | | |
| 10- With regards to using PRO scores in the management of patients with LBP, I expect a good outcome (e.g. better patient-clinician communication, quality of care, better patient’s health outcomes, etc…) | 3 (17%) | 8 (44%) | 7 (39%) |  |  |
| 11- With regards to using PRO scores in the management of patients with LBP, I am optimistic about the benefits for patients | 1 (6%) | 12 (67%) | 3 (25%) | 1 (6%) | 1 (6%) |
| Beliefs about Consequences | | | | | |
| 12- Using PRO scores in the management of patients with LBP is useful and has many advantages for patients | 2 (11%) | 11 (61%) | 4 (22%) | 1 (6%) |  |
| 13- If I do not use PRO scores in the management of patients with LBP, I believe my ability to improve the health of my patients is limited | 1 (6%) | 5 (28%) | 6 (33%) | 4 (22%) | 2 (13%) |
| Reinforcement | | | | | |
| 14- I would use PRO scores every time in the management of patients with LBP if the rewards were greater compared to when I only use clinical measures (e.g. of rewards: better patient satisfaction, less patient discomfort, etc.) | 2 (13%) | 10 (56%) | 5 (28%) | 1 (6%) |  |
| 15- I feel like I am making a positive difference in the quality of patients’ care when I use PRO scores in the management of patients with LBP | 1 (6%) | 5 (28%) | 8 (44%) | 4 (22%) |  |
| Intentions | | | | | |
| 16- I will definitely use PRO scores in the management of patients with LBP in the next three months | 2 (13%) | 10 (56%) | 6 (33%) |  |  |
| 17- I have a strong intention to use PRO scores in the management of patients with LBP in the next three months | 2 (13%) | 10 (56%) | 6 (33%) |  |  |
| Goals | | | | | |
| 18- I have a clear plan of how I will use PRO scores in the management of patients with LBP | 2 (13%) | 6 (33%) | 5 (28%) | 4 (22%) | 1 (6%) |
| 19- Using PRO scores in the management of patients with LBP is more important and prioritized compared to only using clinical outcomes (e.g. straight leg raising, lower extremity strength, sensory test, six minutes walking test, psychological assessment) in achieving the desired patient outcome | 1 (13%) | 2 (13%) | 6 (33%) | 6 (33%) | 3 (17%) |
| Memory/Attention/ and Decision Processes | | | | | |
| 20- It is easy to use PRO scores to help in making treatment decision | 1 (6%) | 6 (33%) | 5 (28%) | 5 (28%) | 1 (6%) |
| 21- I will not forget to use PRO scores in the management of patients with LBP | 1 (6%) | 5 (28%) | 8 (44%) | 4 (22%) |  |
| Environmental Context and Resources | | | | | |
| 22- The resources that I need to help me use PRO scores in the management of patients with LBP are available | 1 (6%) | 7 (39%) | 6 (33%) | 4 (22%) |  |
| 23- There is enough time to use PRO scores in the management of patients with LBP | 1 (6%) | 5 (28%) | 6 (33%) | 6 (33%) |  |
| Social Influences | | | | | |
| 24- The views of my patients influence my decision to use PRO scores in the management of patients with LBP (rescaled) |  | 2 (13%) | 10 (56%) | 6 (33%) |  |
| 25- There is a good collaboration and communication between the interdisciplinary team members that facilitates using PRO scores in the management of patients with LBP | 4 (22%) | 4 (22%) | 8 (44%) | 2 (13%) |  |
| 26- I may consult other people for their opinion regarding the need for using PRO scores in the management of patients with LBP | 2 (11%) | 4 (22%) | 9 (50%) | 3 (17%) |  |
| Emotion | | | | | |
| 27- I generally feel nervous with regard to using PRO scores in the management of patients with LBP (rescaled) | 7 (39%) | 7 (39%) | 4 (22%) |  |  |
| 28- I generally feel inspired to use PRO scores in the management of patients with LBP | 1 (6%) | 6 (33%) | 4 (22%) | 7 (39%) |  |
| Behavioral Regulation | | | | | |
| 29- I assess patient’s motivation to complete PRO | 1 (6%) | 5 (29%) | 6 (33%) | 4 (22%) | 2 (11%) |
| 30- Using PRO scores in the management of patients with LBP is something I do automatically | 1 (6%) | 8 (44%) | 4 (22%) | 4 (22%) | 1 (6%) |
