# Supplementary material for: Developing theory-informed knowledge translation strategies to facilitate the use of patient-reported outcome measures in interdisciplinary low back pain clinical practices in Quebec: mixed methods study
Source: BMC Health Serv Res. 2020 Aug 25;20:789. doi: 10.1186/s12913-020-05616-5 (PMC7445906; doi:10.1186/s12913-020-05616-5)
Supplement: Supplementary file 2 — Additional file 2. PROMs Survey. [file 12913_2020_5616_MOESM2_ESM.docx]

**Additional File 2: PROMs Survey**

**Use of Patient Reported Outcomes in clinical practice Survey (English)**

The following questions are intended to evaluate your views on the management of patients with chronic pain in relation to the use of patient reported outcomes (PROs) within your clinical practice.

The purpose of this questionnaire is to better understand your experience with using PROs to manage individuals with LBP. The term PROs refers to “any report of the status of a patient’s health condition that comes directly from the patient, without interpretation of the patient’s response by a clinician or anyone else”.

Your responses will help us to identify solutions to support clincians’ with using patient reported outcomes in clinical practice

As you move through the survey, please list any additional thoughts you have about the questions posed when asked to do so.

1. **I am aware of the objectives of using PRO scores in the management of patients with LBP**

| Strongly Agree | Agree | Neutral | Disagree | Strongly Disagree |
| --- | --- | --- | --- | --- |
| _1_ | _2_ | _3_ | _4_ | _5_ |

1. **I have sufficient knowledge about how to apply PRO scores in the management of patients with LBP**

| Strongly Agree | Agree | Neutral | Disagree | Strongly Disagree |
| --- | --- | --- | --- | --- |
| _1_ | _2_ | _3_ | _4_ | _5_ |

1. **I know how to interpret PRO scores in the management of patients with LBP**

| Strongly Agree | Agree | Neutral | Disagree | Strongly Disagree |
| --- | --- | --- | --- | --- |
| _1_ | _2_ | _3_ | _4_ | _5_ |

**What information do you believe is necessary for a clinician to be able to use PRO scores in the management of patients with LBP?**

_______________________________________________________________________________________________________________________________________________________________________________________________________________________________________________________________

**Please provide additional comments to explain your responses to the questions 1-3:**

_______________________________________________________________________________________________________________________________________________________________________________________________________________________________________________________________

1. **I would use PRO scores every time in the management of patients with LBP if the rewards were greater compared to when I only use clinical measures (e.g. of rewards: better patient satisfaction, less patient discomfort, etc.)**

| Strongly Agree | Agree | Neutral | Disagree | Strongly Disagree |
| --- | --- | --- | --- | --- |
| _1_ | _2_ | _3_ | _4_ | _5_ |

1. **I have the skills to interpret PRO scores in the management of patients with LBP**

| Strongly Agree | Agree | Neutral | Disagree | Strongly Disagree |
| --- | --- | --- | --- | --- |
| _1_ | _2_ | _3_ | _4_ | _5_ |

1. **I need to acquire new skills to be able to use PRO scores in the management of patients with LBP**

| Strongly Agree | Agree | Neutral | Disagree | Strongly Disagree |
| --- | --- | --- | --- | --- |
| _1_ | _2_ | _3_ | _4_ | _5_ |

**What new skills do you feel you need to acquire to be able to use PRO scores for individual patient management of LBP?**

_______________________________________________________________________________________________________________________________________________________________________________________________________________________________________________________________

1. **I think my role as …… should include using PRO scores for individual patient management of LBP**

| Strongly Agree | Agree | Neutral | Disagree | Strongly Disagree |
| --- | --- | --- | --- | --- |
| _1_ | _2_ | _3_ | _4_ | _5_ |

1. **With regards to using PRO scores in the management of patients with LBP, I expect a good outcome (e.g. better patient-clinician communication, quality of care, better patient’s health outcomes, etc…)**

| Strongly Agree | Agree | Neutral | Disagree | Strongly Disagree |
| --- | --- | --- | --- | --- |
| _1_ | _2_ | _3_ | _4_ | _5_ |

**Please provide additional comments to explain your responses to the questions 4-8:**

_______________________________________________________________________________________________________________________________________________________________________________________________________________________________________________________________

1. **I feel like I am making a positive difference in the quality of patients’ care when I use PRO scores in the management of patients with LBP**

| Strongly Agree | Agree | Neutral | Disagree | Strongly Disagree |
| --- | --- | --- | --- | --- |
| _1_ | _2_ | _3_ | _4_ | _5_ |

1. **I am confident that I can use PRO scores in the management of patients with LBP**

| Strongly Agree | Agree | Neutral | Disagree | Strongly Disagree |
| --- | --- | --- | --- | --- |
| _1_ | _2_ | _3_ | _4_ | _5_ |

**How easy or difficult is using PRO scores in the management of patients with LBP? What problems or barriers have you encountered using PRO scores for the management of patients with LBP?**

_______________________________________________________________________________________________________________________________________________________________________________________________________________________________________________________________

1. **With regards to using PRO scores in the management of patients with LBP, I am optimistic about the benefits for patients**

| Strongly Agree | Agree | Neutral | Disagree | Strongly Disagree |
| --- | --- | --- | --- | --- |
| _1_ | _2_ | _3_ | _4_ | _5_ |

**Please provide additional comments to explain your responses to the questions 9-11:**

_______________________________________________________________________________________________________________________________________________________________________________________________________________________________________________________________

1. **I generally feel nervous with regard to using PRO scores in the management of patients with LBP**

| Strongly Agree | Agree | Neutral | Disagree | Strongly Disagree |
| --- | --- | --- | --- | --- |
| _1_ | _2_ | _3_ | _4_ | _5_ |

1. **Using PRO scores in the management of patients with LBP is useful and has many advantages for patients**

| Strongly Agree | Agree | Neutral | Disagree | Strongly Disagree |
| --- | --- | --- | --- | --- |
| _1_ | _2_ | _3_ | _4_ | _5_ |

**What are the benefits of using PRO scores for patient management of LBP?**

_______________________________________________________________________________________________________________________________________________________________________________________________________________________________________________________________

**What are the potential disadvantages of using PRO scores in the management of patients with LBP?**

_______________________________________________________________________________________________________________________________________________________________________________________________________________________________________________________________

1. **My role in using PRO scores in the management of patients with LBP are clearly defined for me**

| Strongly Agree | Agree | Neutral | Disagree | Strongly Disagree |
| --- | --- | --- | --- | --- |
| _1_ | _2_ | _3_ | _4_ | _5_ |

1. **I am comfortable with using PRO scores in the management of patients with LBP**

| Strongly Agree | Agree | Neutral | Disagree | Strongly Disagree |
| --- | --- | --- | --- | --- |
| _1_ | _2_ | _3_ | _4_ | _5_ |

**Please provide additional comments to explain your responses to the questions 12-15:**

_______________________________________________________________________________________________________________________________________________________________________________________________________________________________________________________________

1. **I will definitely use PRO scores in the management of patients with LBP in the next three months**

| Strongly Agree | Agree | Neutral | Disagree | Strongly Disagree |
| --- | --- | --- | --- | --- |
| _1_ | _2_ | _3_ | _4_ | _5_ |

1. **I have a clear plan of how I will use PRO scores in the management of patients with LBP**

| Strongly Agree | Agree | Neutral | Disagree | Strongly Disagree |
| --- | --- | --- | --- | --- |
| _1_ | _2_ | _3_ | _4_ | _5_ |

1. **It is easy to use PRO scores to help in making treatment decision**

| Strongly Agree | Agree | Neutral | Disagree | Strongly Disagree |
| --- | --- | --- | --- | --- |
| _1_ | _2_ | _3_ | _4_ | _5_ |

**Please provide additional comments to explain your responses to the questions 16-18:**

_______________________________________________________________________________________________________________________________________________________________________________________________________________________________________________________________

1. **If I do not use PRO scores in the management of patients with LBP, I believe my ability to improve the health of my patients is limited**

| Strongly Agree | Agree | Neutral | Disagree | Strongly Disagree |
| --- | --- | --- | --- | --- |
| _1_ | _2_ | _3_ | _4_ | _5_ |

1. **The resources that I need to help me use PRO scores in the management of patients with LBP are available**

| Strongly Agree | Agree | Neutral | Disagree | Strongly Disagree |
| --- | --- | --- | --- | --- |
| _1_ | _2_ | _3_ | _4_ | _5_ |

1. **There is enough time to use PRO scores in the management of patients with LBP**

| Strongly Agree | Agree | Neutral | Disagree | Strongly Disagree |
| --- | --- | --- | --- | --- |
| _1_ | _2_ | _3_ | _4_ | _5_ |

**Are there any factors *(e.g. motivation, availability of patients’ scores, enough time, etc…)* in your practice likely to help you use PRO scores in the management of patients with LBP?**

_______________________________________________________________________________________________________________________________________________________________________________________________________________________________________________________________

**Are there any factors *(e.g. lack of knowledge, lack of time, lack of access to patients’ scores, etc…)* in your practice likely to prevent you using PRO scores in the management of patients with LBP?**

_______________________________________________________________________________________________________________________________________________________________________________________________________________________________________________________________

**Please provide additional comments to explain your responses to the questions 19-21:**

_______________________________________________________________________________________________________________________________________________________________________________________________________________________________________________________________

1. **I will not forget to use PRO scores in the management of patients with LBP**

| Strongly Agree | Agree | Neutral | Disagree | Strongly Disagree |
| --- | --- | --- | --- | --- |
| _1_ | _2_ | _3_ | _4_ | _5_ |

1. **The views of my patients influence my decision to use PRO scores in the management of patients with LBP**

| Strongly Agree | Agree | Neutral | Disagree | Strongly Disagree |
| --- | --- | --- | --- | --- |
| _1_ | _2_ | _3_ | _4_ | _5_ |

1. **Using PRO scores in the management of patients with LBP is more important and prioritized compared to only using clinical outcomes (e.g. straight leg raising, lower extremity strength, sensory test, six minutes walking test, psychological assessment) in achieving the desired patient outcome**

| Strongly Agree | Agree | Neutral | Disagree | Strongly Disagree |
| --- | --- | --- | --- | --- |
| _1_ | _2_ | _3_ | _4_ | _5_ |

1. **There is a good collaboration and communication between the interdisciplinary team members that facilitates using PRO scores in the management of patients with LBP**

| Strongly Agree | Agree | Neutral | Disagree | Strongly Disagree |
| --- | --- | --- | --- | --- |
| _1_ | _2_ | _3_ | _4_ | _5_ |

**Please provide additional comments to explain your responses to the questions 22-25:**

_______________________________________________________________________________________________________________________________________________________________________________________________________________________________________________________________

1. **I generally feel inspired to use PRO scores in the management of patients with LBP**

| Strongly Agree | Agree | Neutral | Disagree | Strongly Disagree |
| --- | --- | --- | --- | --- |
| _1_ | _2_ | _3_ | _4_ | _5_ |

1. **I assess patient’s motivation to complete PROs**

| Strongly Agree | Agree | Neutral | Disagree | Strongly Disagree |
| --- | --- | --- | --- | --- |
| _1_ | _2_ | _3_ | _4_ | _5_ |

1. **Using PRO scores in the management of patients with LBP is something I do automatically**

| Strongly Agree | Agree | Neutral | Disagree | Strongly Disagree |
| --- | --- | --- | --- | --- |
| _1_ | _2_ | _3_ | _4_ | _5_ |

1. **I have a strong intention to use PRO scores in the management of patients with LBP in the next three months**

| Strongly Agree | Agree | Neutral | Disagree | Strongly Disagree |
| --- | --- | --- | --- | --- |
| _1_ | _2_ | _3_ | _4_ | _5_ |

1. **I may consult other people for their opinion regarding the need for using PRO scores in the management of patients with LBP**

| Strongly Agree | Agree | Neutral | Disagree | Strongly Disagree |
| --- | --- | --- | --- | --- |
| _1_ | _2_ | _3_ | _4_ | _5_ |

**Please provide additional comments to explain your responses to the questions 26-30:**

_______________________________________________________________________________________________________________________________________________________________________________________________________________________________________________________________

**Is there anything else you want to add regarding the use of PRO scores in the management of patients with LBP?**

_______________________________________________________________________________________________________________________________________________________________________________________________________________________________________________________________

**Utilisation en pratique clinique des ré**sultats signalés par les patients (Patient Reported Outcomes [PRO])

Les questions suivantes ont pour but d’évaluer votre point de vue concernant la prise en charge des patients atteints de douleur chronique relativement à l’utilisation de résultats signalés par les patients (Patient Reported Outcomes [PRO]) dans votre pratique clinique.
Le but de ce questionnaire est de nous aider à mieux comprendre votre expérience avec l’utilisation de PRO dans la prise en charge des individus souffrant de douleur chronique. L’expression « résultats signalés par les patients » ou « PRO » fait référence à « tout rapport de l’état de santé d’un patient qui vient directement du patient, sans interprétation de la réponse du patient par un médecin ou quelqu’un d’autre ». Souvent, ces résultats sont recueillis par des questionnaires que les patients s’auto-administrent.

Vos réponses nous permettront de trouver des solutions pour aider les cliniciens à utiliser des PRO dans la pratique clinique

Tout au long du questionnaire, nous vous demanderons de fournir des commentaires supplémentaires concernant les questions posées.

Le masculin est utilisé pour alléger le texte.

1. **Je suis conscient des objectifs de l’utilisation des scores PRO dans la prise en charge des patients atteints de douleur chronique.**

| Tout à fait d’accord | D’accord | Ni d’accord ni en désaccord | En désaccord | Totalement en désaccord |
| --- | --- | --- | --- | --- |
| _1_ | _2_ | _3_ | _4_ | _5_ |

1. **J’ai suffisamment de connaissances sur la façon d’appliquer les scores PRO dans la prise en charge des patients atteints de douleur chronique.**

| Tout à fait d’accord | D’accord | Ni d’accord ni en désaccord | En désaccord | Totalement en désaccord |
| --- | --- | --- | --- | --- |
| _1_ | _2_ | _3_ | _4_ | _5_ |

1. **Je sais comment interpréter les scores PRO dans la prise en charge des patients atteints de douleur chronique.**

| Tout à fait d’accord | D’accord | Ni d’accord ni en désaccord | En désaccord | Totalement en désaccord |
| --- | --- | --- | --- | --- |
| _1_ | _2_ | _3_ | _4_ | _5_ |

**Quelles informations considérez-vous comme nécessaires pour qu’un clinicien soit en mesure d’utiliser les scores PRO dans la prise en charge des patients atteints de douleur chronique?**

_______________________________________________________________________________________________________________________________________________________________________________________________________________________________________________________________

**Veuillez fournir des commentaires supplémentaires pour expliquer vos réponses aux questions 1 à 3.**

_______________________________________________________________________________________________________________________________________________________________________________________________________________________________________________________________

1. **J’utiliserais toujours les scores PRO dans la prise en charge des patients atteints de douleur chronique si les avantages étaient plus grands que lorsque j’utilise seulement des mesures cliniques (exemples d’avantages : plus grande satisfaction du patient, moins d’inconfort pour le patient, etc.).**

| Tout à fait d’accord | D’accord | Ni d’accord ni en désaccord | En désaccord | Totalement en désaccord |
| --- | --- | --- | --- | --- |
| _1_ | _2_ | _3_ | _4_ | _5_ |

1. **Je possède les compétences nécessaires pour interpréter les scores PRO dans la prise en charge des patients atteints de douleur chronique.**

| Tout à fait d’accord | D’accord | Ni d’accord ni en désaccord | En désaccord | Totalement en désaccord |
| --- | --- | --- | --- | --- |
| _1_ | _2_ | _3_ | _4_ | _5_ |

1. **J’ai besoin d’acquérir de nouvelles compétences pour pouvoir utiliser des scores PRO dans la prise en charge des patients atteints de douleur chronique.**

| Tout à fait d’accord | D’accord | Ni d’accord ni en désaccord | En désaccord | Totalement en désaccord |
| --- | --- | --- | --- | --- |
| _1_ | _2_ | _3_ | _4_ | _5_ |

**Quelles nouvelles compétences sentez-vous le besoin d’acquérir pour être en mesure d’utiliser des scores PRO dans la prise en charge des patients atteints de douleur chronique?**

_______________________________________________________________________________________________________________________________________________________________________________________________________________________________________________________________

1. **Je pense que mon rôle en tant que professionnel de la santé devrait inclure l’utilisation de scores PRO pour la prise en charge de certains patients atteints de douleur chronique.**

| Tout à fait d’accord | D’accord | Ni d’accord ni en désaccord | En désaccord | Totalement en désaccord |
| --- | --- | --- | --- | --- |
| _1_ | _2_ | _3_ | _4_ | _5_ |

1. **En ce qui concerne l’utilisation de scores PRO dans la prise en charge des patients atteints de douleur chronique, je m’attends à de meilleurs résultats (p. ex., une meilleure communication entre le patient et le clinicien, une meilleure qualité des soins, de meilleurs résultats de santé des patients, etc.).**

| Tout à fait d’accord | D’accord | Ni d’accord ni en désaccord | En désaccord | Totalement en désaccord |
| --- | --- | --- | --- | --- |
| _1_ | _2_ | _3_ | _4_ | _5_ |

**Veuillez fournir des commentaires supplémentaires pour expliquer vos réponses aux questions 4 à 8.**

_______________________________________________________________________________________________________________________________________________________________________________________________________________________________________________________________

1. **J’ai l’impression de contribuer positivement à améliorer la qualité des soins aux patients quand j’utilise des scores PRO dans la prise en charge des patients atteints de douleur chronique.**

| Tout à fait d’accord | D’accord | Ni d’accord ni en désaccord | En désaccord | Totalement en désaccord |
| --- | --- | --- | --- | --- |
| _1_ | _2_ | _3_ | _4_ | _5_ |

1. **Je suis persuadé de pouvoir utiliser des scores PRO dans la prise en charge des patients atteints de douleur chronique.**

| Tout à fait d’accord | D’accord | Ni d’accord ni en désaccord | En désaccord | Totalement en désaccord |
| --- | --- | --- | --- | --- |
| _1_ | _2_ | _3_ | _4_ | _5_ |

**L’utilisation de scores PRO dans la prise en charge des patients atteints de douleur chronique est-elle facile ou difficile? À quels problèmes ou obstacles vous êtes-vous heurtés en utilisant les scores PRO?**

_______________________________________________________________________________________________________________________________________________________________________________________________________________________________________________________________

1. **Je crois fermement aux avantages pour les patients de l’utilisation de scores PRO dans la prise en charge des patients atteints de douleur chronique.**

| Tout à fait d’accord | D’accord | Ni d’accord ni en désaccord | En désaccord | Totalement en désaccord |
| --- | --- | --- | --- | --- |
| _1_ | _2_ | _3_ | _4_ | _5_ |

**Veuillez fournir des commentaires supplémentaires pour expliquer vos réponses aux questions 9 à 11.** _______________________________________________________________________________________________________________________________________________________________________________________________________________________________________________________________

1. **En général, l’utilisation des scores PRO dans la prise en charge des patients atteints de douleur chronique me rend nerveux.**

| Tout à fait d’accord | D’accord | Ni d’accord ni en désaccord | En désaccord | Totalement en désaccord |
| --- | --- | --- | --- | --- |
| _1_ | _2_ | _3_ | _4_ | _5_ |

1. **Selon moi, l’utilisation de scores PRO dans la prise en charge des patients atteints de douleur chronique est utile et présente de nombreux avantages pour les patients.**

| Tout à fait d’accord | D’accord | Ni d’accord ni en désaccord | En désaccord | Totalement en désaccord |
| --- | --- | --- | --- | --- |
| _1_ | _2_ | _3_ | _4_ | _5_ |

**Quels sont les avantages de l’utilisation des scores PRO dans la prise en charge des patients atteints de douleur chronique?** _______________________________________________________________________________________________________________________________________________________________________________________________________________________________________________________________

**Quels sont les désavantages potentiels de l’utilisation des scores PRO dans la prise en charge des patients atteints de douleur chronique?** _______________________________________________________________________________________________________________________________________________________________________________________________________________________________________________________________

1. **Mon rôle dans l’utilisation des scores PRO dans la prise en charge des patients atteints de douleur chronique a été clairement défini.**

| Tout à fait d’accord | D’accord | Ni d’accord ni en désaccord | En désaccord | Totalement en désaccord |
| --- | --- | --- | --- | --- |
| _1_ | _2_ | _3_ | _4_ | _5_ |

1. **Je suis à l’aise quant à l’utilisation des scores PRO dans la prise en charge des patients atteints de douleur chronique.**

| Tout à fait d’accord | D’accord | Ni d’accord ni en désaccord | En désaccord | Totalement en désaccord |
| --- | --- | --- | --- | --- |
| _1_ | _2_ | _3_ | _4_ | _5_ |

**Veuillez fournir des commentaires supplémentaires pour expliquer vos réponses aux questions 12 à 15.** _______________________________________________________________________________________________________________________________________________________________________________________________________________________________________________________________

1. **Je vais certainement utiliser des scores PRO dans la prise en charge des patients atteints de douleur chronique dans les trois prochains mois.**

| Tout à fait d’accord | D’accord | Ni d’accord ni en désaccord | En désaccord | Totalement en désaccord |
| --- | --- | --- | --- | --- |
| _1_ | _2_ | _3_ | _4_ | _5_ |

1. **J’ai un plan clair de la façon dont je vais utiliser des scores PRO dans la prise en charge des patients atteints de douleur chronique.**

| Tout à fait d’accord | D’accord | Ni d’accord ni en désaccord | En désaccord | Totalement en désaccord |
| --- | --- | --- | --- | --- |
| _1_ | _2_ | _3_ | _4_ | _5_ |

1. **Je trouve facile d’utiliser des scores PRO afin de faciliter la prise de décision concernant le traitement.**

| Tout à fait d’accord | D’accord | Ni d’accord ni en désaccord | En désaccord | Totalement en désaccord |
| --- | --- | --- | --- | --- |
| _1_ | _2_ | _3_ | _4_ | _5_ |

**Veuillez fournir des commentaires supplémentaires pour expliquer vos réponses aux questions 16 à 18.** _______________________________________________________________________________________________________________________________________________________________________________________________________________________________________________________________

1. **Si je n’utilise pas de scores PRO dans la prise en charge des patients atteints de douleur chronique, je crois que ma capacité d’améliorer la santé de mes patients sera limitée.**

| Tout à fait d’accord | D’accord | Ni d’accord ni en désaccord | En désaccord | Totalement en désaccord |
| --- | --- | --- | --- | --- |
| _1_ | _2_ | _3_ | _4_ | _5_ |

1. **J’ai accès aux ressources dont j’ai besoin pour m’aider à utiliser des scores PRO dans la prise en charge des patients atteints de douleur chronique.**

| Tout à fait d’accord | D’accord | Ni d’accord ni en désaccord | En désaccord | Totalement en désaccord |
| --- | --- | --- | --- | --- |
| _1_ | _2_ | _3_ | _4_ | _5_ |

**Y a-t-il des facteurs (p. ex., motivation, disponibilité des scores des patients, temps suffisant, etc.) dans votre pratique qui pourraient vous aider à utiliser des scores PRO dans la prise en charge des patients atteints de douleur chronique?** _______________________________________________________________________________________________________________________________________________________________________________________________________________________________________________________________

**Y a-t-il des facteurs (p. ex., manque de connaissances, manque de temps, manque d’accès aux scores des patients, etc.) dans votre pratique qui pourraient vous empêcher d’utiliser des scores PRO dans la prise en charge des patients atteints de douleur chronique?** _______________________________________________________________________________________________________________________________________________________________________________________________________________________________________________________________

1. **J’ai assez de temps pour utiliser des scores PRO dans la prise en charge des patients atteints de douleur chronique.**

| Tout à fait d’accord | D’accord | Ni d’accord ni en désaccord | En désaccord | Totalement en désaccord |
| --- | --- | --- | --- | --- |
| _1_ | _2_ | _3_ | _4_ | _5_ |

1. **Je ne vais pas oublier d’utiliser des scores PRO dans la prise en charge des patients atteints de douleur chronique.**

| Tout à fait d’accord | D’accord | Ni d’accord ni en désaccord | En désaccord | Totalement en désaccord |
| --- | --- | --- | --- | --- |
| _1_ | _2_ | _3_ | _4_ | _5_ |

**Veuillez fournir des commentaires supplémentaires pour expliquer vos réponses aux questions 19 à 22.** _______________________________________________________________________________________________________________________________________________________________________________________________________________________________________________________________

1. **L’opinion de mes patients influe sur ma décision d’utiliser des scores PRO dans la prise en charge des patients atteints de douleur chronique.**

| Tout à fait d’accord | D’accord | Ni d’accord ni en désaccord | En désaccord | Totalement en désaccord |
| --- | --- | --- | --- | --- |
| _1_ | _2_ | _3_ | _4_ | _5_ |

1. **Pour moi, l’utilisation de scores PRO dans la prise en charge des patients atteints de douleur chronique doit être priorisée et est plus importante que la seule l’utilisation de résultats cliniques (p. ex. élévation de la jambe tendue, force musculaire du membre inférieur, test sensoriel, test de marche de six minutes, évaluation psychologique, etc.) pour atteindre le résultat désiré par le patient.**

| Tout à fait d’accord | D’accord | Ni d’accord ni en désaccord | En désaccord | Totalement en désaccord |
| --- | --- | --- | --- | --- |
| _1_ | _2_ | _3_ | _4_ | _5_ |

1. **La collaboration et la communication entre les membres de l’équipe interdisciplinaire sont bonnes, ce qui facilite l’utilisation des scores PRO dans la prise en charge des patients atteints de douleur chronique.**

| Tout à fait d’accord | D’accord | Ni d’accord ni en désaccord | En désaccord | Totalement en désaccord |
| --- | --- | --- | --- | --- |
| _1_ | _2_ | _3_ | _4_ | _5_ |

1. **En général, j’ai envie d’utiliser des scores PRO dans la prise en charge des patients atteints de douleur chronique.**

| Tout à fait d’accord | D’accord | Ni d’accord ni en désaccord | En désaccord | Totalement en désaccord |
| --- | --- | --- | --- | --- |
| _1_ | _2_ | _3_ | _4_ | _5_ |

**Veuillez fournir des commentaires supplémentaires pour expliquer vos réponses aux questions 22 à 26.** _______________________________________________________________________________________________________________________________________________________________________________________________________________________________________________________________

1. **J’évalue la motivation du patient à remplir des questionnaires PRO.**

| Tout à fait d’accord | D’accord | Ni d’accord ni en désaccord | En désaccord | Totalement en désaccord |
| --- | --- | --- | --- | --- |
| _1_ | _2_ | _3_ | _4_ | _5_ |

1. **L’utilisation de scores PRO dans la prise en charge des patients atteints de douleur chronique est quelque chose que je fais automatiquement.**

| Tout à fait d’accord | D’accord | Ni d’accord ni en désaccord | En désaccord | Totalement en désaccord |
| --- | --- | --- | --- | --- |
| _1_ | _2_ | _3_ | _4_ | _5_ |

1. **J’ai la ferme intention d’utiliser des scores PRO dans la prise en charge des patients atteints de douleur chronique dans les trois prochains mois.**

| Tout à fait d’accord | D’accord | Ni d’accord ni en désaccord | En désaccord | Totalement en désaccord |
| --- | --- | --- | --- | --- |
| _1_ | _2_ | _3_ | _4_ | _5_ |

1. **Il se peut que je consulte d’autres personnes pour obtenir leur opinion sur la nécessité d’utiliser des scores PRO dans la prise en charge des patients atteints de douleur chronique.**

| Tout à fait d’accord | D’accord | Ni d’accord ni en désaccord | En désaccord | Totalement en désaccord |
| --- | --- | --- | --- | --- |
| _1_ | _2_ | _3_ | _4_ | _5_ |

**Veuillez fournir des commentaires supplémentaires pour expliquer vos réponses aux questions 26 à 30.** _______________________________________________________________________________________________________________________________________________________________________________________________________________________________________________________________

**Y a-t-il autre chose que vous voulez ajouter au sujet de l’utilisation de scores PRO dans la prise en charge des patients atteints de douleur chronique? Veuillez, s’il vous plaît, l’écrire ci-dessous.** _______________________________________________________________________________________________________________________________________________________________________________________________________________________________________________________________
