# Supplementary material for: Developing theory-informed knowledge translation strategies to facilitate the use of patient-reported outcome measures in interdisciplinary low back pain clinical practices in Quebec: mixed methods study
Source: BMC Health Serv Res. 2020 Aug 25;20:789. doi: 10.1186/s12913-020-05616-5 (PMC7445906; doi:10.1186/s12913-020-05616-5)

**Additional File 3: Patient Reported Outcome Measures (PROMs) KT intervention**

**Workshop* (5 hours)**

- Introducing the concept of PROMs (2 hrs)
- Definition of PROMs: *defined as “any report of the status of a patient’s health condition that comes directly from the patient, without interpretation of the patient’s response by a clinician or anyone else”*
- Effectiveness of using PROMs
- *Inﬂuencing communication (patient-clinician, clinician-clinician, clinician-relatives, patient-relatives)*
- *Uncovering patients’ problems, such as psychological and functional problems*
- *Monitoring response to treatment*
- *Providing information about the impact of prescribed treatments*
- *Informing clinical decision-making*
- *Identifying shortages in the current care provided*
- *Feeding back clinicians information on HRQOL from PROM scores will prompt clinician-patient discussion of HRQOL issues and allow for mutual input on treatment goal setting*
- The role of clinicians in using PROMs in the clinical practice – Case Studies
- *Reviewing patients’ scores using PROM scores report*
- *Discussion the health issues with patients*
- *Taking actions to address the patient issues*
- Introducing the most frequent used questionnaires in LBP
- *Brief Pain Inventory (BPI)*
- *Oswestry Disability Index*
- *Hospital Anxiety and Depression Scale*
- *SF-36*
- Scoring algorithm and interpretation

**--------- Break (15 min) --------**

- Introducing the PROMs scores report** (1 hr)
- Introduce its components
- *Patients Scores overtime*
- *Interpretation of the scores*
- *Changes of patients scores based on minimal clinical important difference*
- *Treatment plan*
- How to use it
- Practice (1 hr)
- Give each clinician different PROM scores report to interpret and use it in making treatment decision

**--------- Break (15 min) --------**

- Discussion, questions, and closing mark (30 – 60 min)

* The workshop will be guided by the research team

**Opinion Leader**

- The opinion leader can be selected by each clinical site and ideally meeting the following criteria:
- opinion leaders will receive training in the selection and application of PROMs.
- They will attend a workshop similar to the aforementioned one, but it will be conducted before the clinicians’ workshop and will integrate a components of coaching.

**PROM Scores Feedback Report****

- The scores report includes
- Patients scores overtime
- Interpretation of the scores
- Minimal clinical important difference information ( Improvement, No change, Deterioration)
- Link patients scores to the intervention

** The following is an example of the PROM scores feedback report


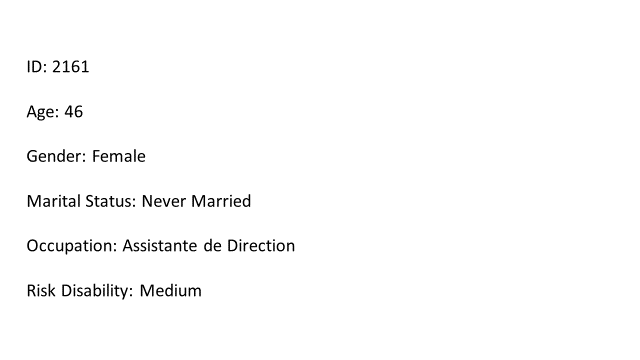


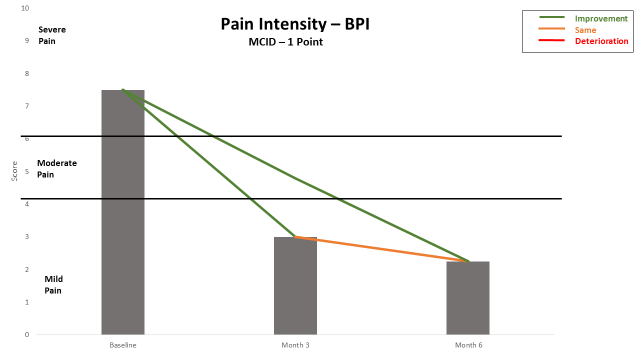


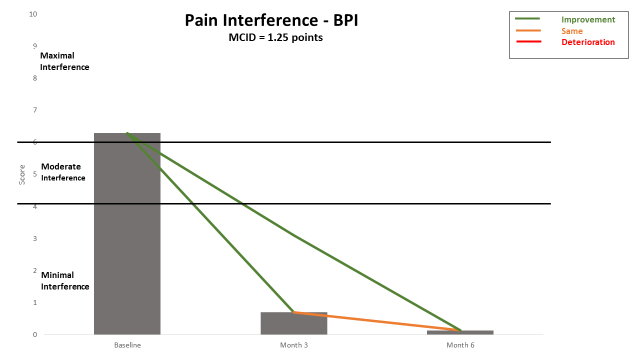


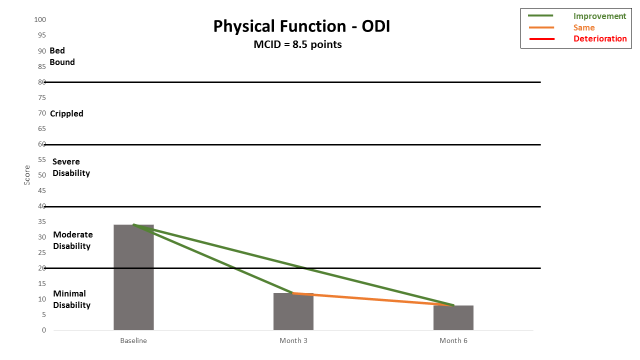


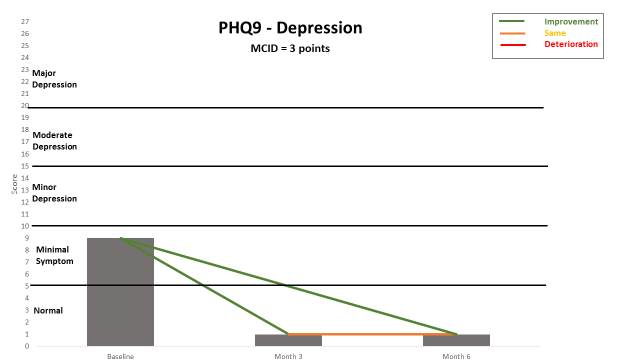


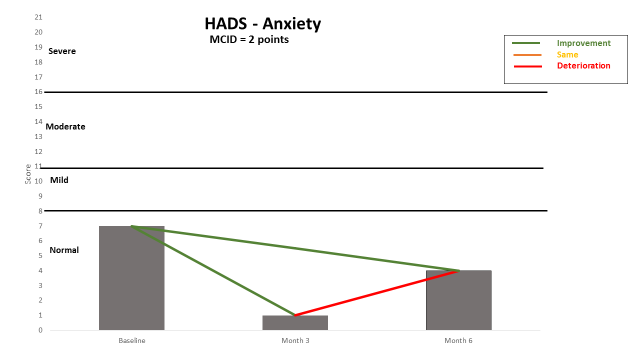


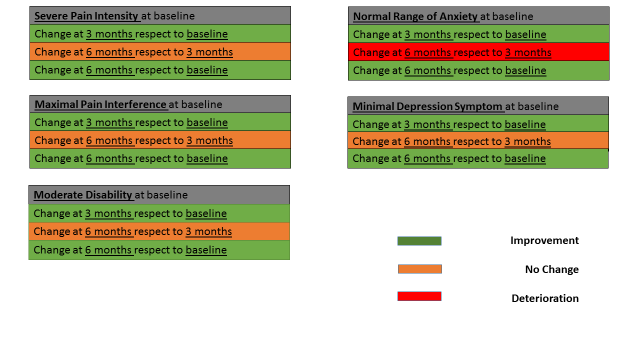


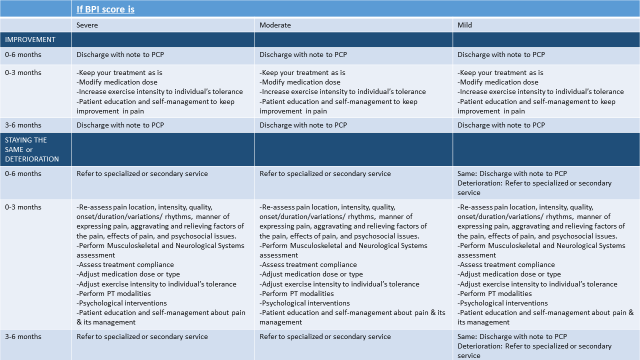


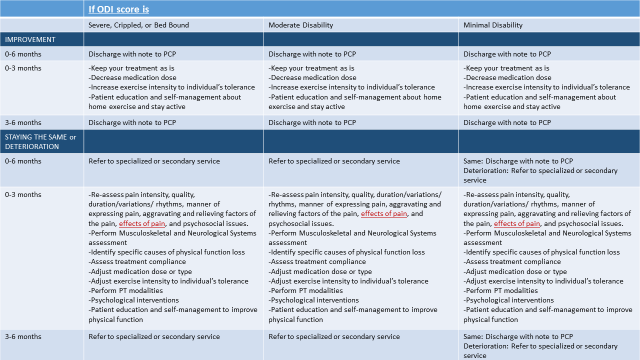


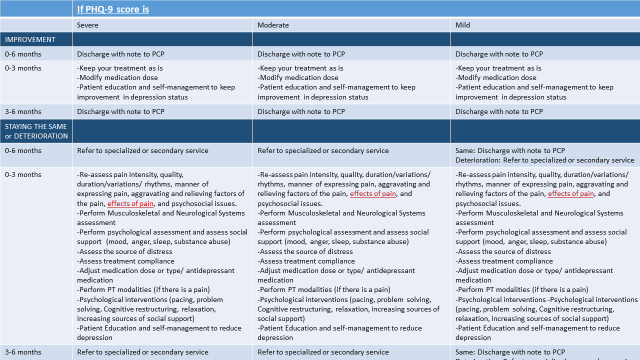


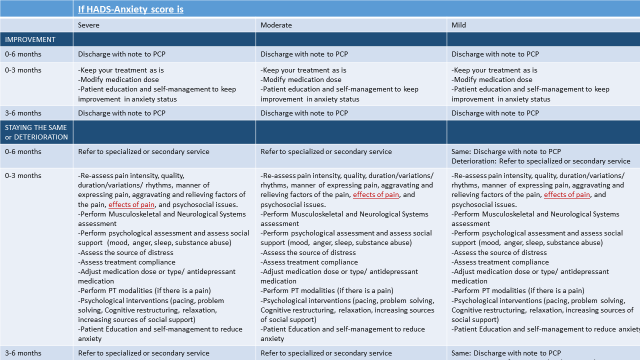

Supplement: Supplementary file 3 — Additional file 3. Patient Reported Outcome Measures (PROMs) KT intervention. [file 12913_2020_5616_MOESM3_ESM.docx]
